# Asymptomatic hyperuricemia is not an independent risk factor for cardiovascular events or overall mortality in the general population of the Busselton Health Study

**DOI:** 10.1186/s12872-016-0421-1

**Published:** 2016-12-15

**Authors:** Johannes Nossent, Warren Raymond, Mark Divitini, Matthew Knuiman

**Affiliations:** 1 School of Medicine & Pharmacology, The University of Western Australia, 35 Stirling Hwy (M503), Perth, 6009 WA Australia; 2 Department of Rheumatology, Sir Charles Gairdner & Osborne Park Hospital Group, Perth, 6009 WA Australia; 3 School of Population Health, The University of Western Australia, Perth, 6009 WA Australia

**Keywords:** Hyperuricemia, Cardiovascular disease, Mortality, Gout

## Abstract

**Background:**

To investigate the impact of uric acid (UA) levels on cardiovascular disease and mortality at a population level.

**Methods:**

Prospective analysis of baseline serum UA measurement and 15 year follow-up data from the Busselton Health Survey (*n* = 4,173), stratified by existence or absence of baseline cardiovascular disease. Outcomes were ascertained from state-wide hospital discharge and mortality registries. Cox regression produced adjusted hazard ratios (HR) for UA level as continuous and categorical (low, medium, high) predictor for cardiovascular events (CVE) and mortality. Gout was defined as a patient’s self-reported history of gout.

**Results:**

After age and gender adjustment each 0.1 mmol/L rise in UA level was associated with increased mortality (HR 1.19, CI 1.04–1.36), cardiovascular mortality (HR 1.27, CI 1.03–1.57) and first CVE (HR 1.28, CI 1.13–1.44) in participants with no history of CVE. Adjustment for behavioural and biomedical risk factors of cardiovascular disease attenuated these associations. Results for participants with a history of CVE and for a subset of 1,632 participants using UA levels (2–6 measurements) averaged over time were similar. The overall prevalence of hyperuricemia was 10.7%. When stratified by history of gout, UA level was significantly associated with increased risk of cardiovascular mortality only in participants with a history of CVE (HR 2.13, CI 1.03–4.43).

**Conclusions:**

Despite the considerable prevalence of hyperuricemia in 10.7% of the population, single or time averaged measures of UA were not independently predictive of incident cardiovascular disease or mortality. Hyperuricemia did associate with an increased risk of cardiovascular death only in participants with gout and existing cardiovascular disease.

**Electronic supplementary material:**

The online version of this article (doi:10.1186/s12872-016-0421-1) contains supplementary material, which is available to authorized users.

## Background

Hyperuricemia is an increasingly prevalent metabolic condition that develops when inherited or acquired conditions decrease the ability of the kidneys to secrete uric acid [1]. Unique to humans who have become incapable of breaking down uric acid (UA), hyperuricemia leads to widespread uric acid deposition in a variety of tissues. Typically, the first clinical presentation of hyperuricemia is the development of gouty arthritis, in which the build-up of monosodium urate (MSU) crystals causes an activation of the NLRP3 inflammasome response releasing IL-1β and IL-18 in an attempt to attract macrophages to remove the birefringent crystals [2]. If left untreated, crystal deposition can occur in multiple joints, and generates a significant inflammatory response. Experimental studies shows that MSU crystals also can deposit in blood vessels walls including coronary arteries where they can induce endothelial dysfunction, oxidative stress, inflammation and platelet activation [3–9]. Hyperuricemia has been associated with arterial hypertension, stroke and heart disease [10–12], but the clinical significance of hyperuricemia as a risk factor for vascular events remains unresolved, leading to ambiguity regarding the need to treat asymptomatic hyperuricemia [9, 13–25].

The Busselton Health Survey (BHS) was designed to examine the longitudinal relationship between health measures and outcomes in a well described Western Australian population cohort [26, 27]. The aim of the current analysis was to investigate whether increased baseline and time averaged UA levels alone or together with a history of gout were risk factors for cardiovascular events and mortality in the adult BHS cohort.

## Methods

### Baseline measurements and follow-up outcome events

Details of the 1994/95 BHS have been described previously [27]. Survey participants completed a comprehensive questionnaire underwent various measurements and a fasting blood sample collection. Smoking history, alcohol consumption, diet, minutes of moderate and vigorous intensity leisure time physical activity per usual week, diabetes and medications were obtained by questionnaire. Physical activity was calculated as (minutes/week of moderate intensity activities) + 2 × (minutes/week of vigorous intensity activity). Alcohol consumption was labelled ‘light’ if consumption was ≤140 g/week and ‘heavy’ if >140 g/week. The composite diet score ranged from 0 to 8, representing the number of healthier diet choices [26]. Body mass index was defined as weight (kg) divided by height (m) squared. Blood pressure was measured using a mercury sphygmomanometer after five minutes rest in a sitting position. Blood tests for this analysis include serum total and HDL cholesterol, triglycerides, C-reactive protein (CRP), creatinine and UA level (by uricase-based spectrophotometry). Renal function was derived from creatinine level, using the MDRD estimated glomerular filtration rate (eGFR) formula. Gout status was defined as self-report of a physician diagnosis of gout.

About half of the participants (1,632) had attended additional surveys before the 1994/95 survey at which serum UA had been assessed. To overcome potential bias from changes in serum UA measurement methods across the multiple surveys we converted the UA values at each survey to Z-scores using the age group and sex specific means and standard deviations for all people in those cross-sectional surveys (each survey had about 4000 participants). For this sub-cohort of 1994/1994 survey participants with multiple UA measurements (range 2–6) we calculated the average UA Z-score across all their measures as well as the average annual change in their UA Z-score, as determined from a linear regression of their multiple UA Z-scores on calendar year of survey.

Outcome events during the 15 year follow-up period to 2010 were ascertained from death records and the WA Hospital Morbidity Collection which records all hospital separations in public and private hospitals in WA. International Classification of Diseases, 9th revision (ICD-9-CM) codes were used for events up to 30 June 1999, and ICD-10-AM codes for subsequent events. Three outcome events were analysed. Time to death from any cause, time to death from cardiovascular diseases (ICD-9 390–459; ICD-10 I00-99 G45), and time to first fatal or non-fatal cardiovascular event defined as a hospital admission with a principal discharge diagnosis of coronary heart disease (ICD-9 410–414; ICD-10 I20–25) stroke (ICD-9 430–437; ICD-10 I60–68 G45), congestive heart failure (ICD-9 428; ICD-10 I50) peripheral arterial disease (ICD-9 440–448; ICD-10 I70–79) or death from cardiovascular disease. A baseline history of cardiovascular events (CVE) was defined as having any hospital admission for non-fatal, cardiovascular diseases (ICD-9 & ICD-9-CM 390–459) during the 15 years before the survey (i.e. 1980 to 1994). Diabetes was based on self-reported doctor–diagnosed diabetes or on diabetes treatment (tablets/insulin) at the survey or a history of hospital admissions (ICD-9 250). The Human Research Ethics Committee of the Department of Health of WA approved the BHS and WA-HMDC record linkage [28].

### Statistical analysis

Variables with skewed distributions were log transformed for use in regression models. Cox regression models for time from 1994/95 survey to first outcome event were used to obtain adjusted hazard ratios for UA level considered both as a continuous variable (in mmol/L and Z-score) and also as a categorical variable (low medium and high). The cut-off levels for low, medium and high (hyperuricemic) UA levels were based on current normal reference ranges defined by the local pathology laboratory (see footnote to Table 1). Two levels of adjustment were used: Model 1 was adjusted for age (as quadratic) and sex and Model 2 was further adjusted for potential confounders. The list of behavioural and biomedical cardiovascular risk factors considered as potential confounders is shown in Table 1. The variables that were at least modestly associated (i.e. *p* < 0.10) with serum UA and with the CVE event outcome (after adjustment for age and sex) were considered as potential confounders in Model 2 and included smoking status, BMI, SBP, hypertension medications, total cholesterol, HDL cholesterol, In(triglycerides), lipid medications, diuretic use, In(CRP) and eGFR. An extension of Model 2 that included the self-reported history of gout and an interaction between history of gout and UA level was used to determine whether the effect of UA on CVE and mortality outcomes differed in people with and without a history of gout. Results from Cox regression models are presented as estimated hazard ratios (with 95% confidence interval and *p*-value) and *p*-values < 0.05 are considered as significant evidence of an effect. Results are presented for the full cohort and also for the sub-cohort with multiple measures of UA. The proportional hazards assumption for UA in relation to outcomes was tested using an interaction between UA and follow-up time and was found to hold. All analyses were performed using SAS 9.4 software (SAS Institute Inc).

**Table 1:** Characteristics of the full cohort by history of CVE at baseline

Characteristic or measure	CVE history = no (*n* = 3,475)	CVE history = yes (*n* = 698)
Male gender (%)	44.3	44.7
Age (years)	47.8 (16.2)	62.1 (14.5)
Smoking		
Never (%)	53.6	49.4
Ex (%)	32.1	43.4
Current (%)	14.3	7.2
Alcohol		
Never (%)	6.8	7.2
Ex (%)	7.9	14.6
Light (%)	61.3	55.9
Mod/Heavy/Unknown (%)	24.1	22.3
Physical activity (mins/week)	275 (363)	206 (306)
Diet score (range 0 to 8)	3.15 (1.51)	3.35 (1.51)
BMI (kg/m^2^)	25.9 (4.1)	26.6 (4.1)
Systolic BP (mm Hg)	122 (17)	131 (21)
Diastolic BP (mm Hg)	74 (10)	76 (11)
Hypertension meds (%)	11.3	44.4
Cholesterol (mmol/L)	5.50 (1.09)	5.82 (1.09)
HDL (mmol/L)	1.40 (0.38)	1.37 (0.43)
Triglycerides (mmol/L)	1.24 (0.81)	1.54 (1.22)
Lipid meds (%)	1.5	8.0
Diabetes (%)	4.7	11.5
Gout history (%)	1.4	3.6
Diuretic use (%)	3.0	11.7
CRP (mg/L)	2.97 (7.73)	3.86 (10.21)
MDRD eGFR (mL/min/1.73 m^2^)	72.3 (13.1)	63.5 (14.4)
UA (mmol/L)	0.3108 (0.0806)	0.3353 (0.0921)
UA z-score	−0.0345 (0.9722)	0.1173 (1.0649)
UA category^a^		
Low (%)	39.6	35.2
Medium (%)	51.1	48.3
High (%)	9.4	16.5
Outcomes		
Follow-up time (years)	15.4 (2.6)	13.6 (4.4)
Deaths	408 (11.7)	244 (35.0)
CVE deaths	155 (4.5)	118 (16.9)
CVE events	461 (13.3)	301 (43.1)

Table shows mean (SD), percent or N (%)

^a^Low (UA ≤ 0.30 in subjects ≥ 46 years, ≤ 0.24 in women < 46 years) Medium (0.30 < UA ≤ 0.42 in subjects ≥ 46 years, 0.24 < UA ≤ 0.36 in women < 46 years)

High (UA > 0.42 in subjects ≥ 46 years, UA > 0.36 in women < 46 years)

## Results

We included 3,475 participants free of CVE and 698 participants with a history of CVE at baseline. The overall risk factor profile is typical of community samples at that time (Table 1) even though baseline age and prevalence of CVD risk factors were higher in the group with a history of CVE. Mean (SD) UA level was 0.31 mmol/L (0.08) with hyperuricemia observed in 9% in the group free of CVE vs. 16.5% in those with known CVE. During fifteen years of follow-up, 461 (13%) of the group free of CVE experienced a first CVE event and 408 (12%) participants of this group died; CVE accounted for approximately one third of fatalities (myocardial infarction 28 other CHD 35, congestive heart failure 10, stroke 38, peripheral vascular disease 12, and other CVE 32). Mortality, CVE death and CVE recurrence rates were roughly three times higher in those with a history of CVE at baseline.

Table 2 shows estimates for the association between baseline UA levels and CVE and mortality in participants free of CVE at baseline. There is a significant association between UA level and all three outcomes in Model 1 (age and sex adjusted). After further adjustment for potential confounders these associations are attenuated and no longer significant (Model 2). In the group with known CVE, there was again a significant association between UA and cardiovascular mortality and cardiovascular events but not all-cause mortality in Model 1, but associations were no longer significant in Model 2 (Additional file 1: Table S1).

**Table 2:** Association between UA level and all-cause mortality, CVE mortality and CVE event in the full cohort without a history of CVE at baseline

UA measure	Model 1^a^		Model 2^a^	
	HR (95% CI)	*p*-value	HR (95% CI)	*p*-value
All-cause mortality				
UA^b^	**1.19 (1.04,1.36)**	**0.010**	1.15 (0.98,1.35)	0.089
UA z-score^b^	**1.15 (1.03,1.27)**	**0.009**	1.12 (0.99,1.27)	0.069
UA category		0.210		0.666
Low	1.00		1.00	
Medium	1.07 (0.86,1.33)	0.553	1.01 (0.79,1.28)	0.939
High	1.31 (0.97,1.79)	0.080	1.15 (0.81,1.65)	0.428
Cardiovascular mortality				
UA^b^	**1.27 (1.03,1.57)**	**0.025**	1.17 (0.91,1.52)	0.225
UA z-score^b^	**1.21 (1.02,1.43)**	**0.025**	1.14 (0.93,1.40)	0.212
UA category		0.539		0.974
Low	1.00		1.00	
Medium	1.16 (0.81,1.67)	0.411	0.99 (0.67,1.47)	0.964
High	1.30 (0.79,2.15)	0.295	1.05 (0.58,1.90)	0.872
Cardiovascular event				
UA^b^	**1.28 (1.13,1.44)**	**<0.001**	1.09 (0.94,1.26)	0.267
UA z-score^b^	**1.19 (1.08,1.31)**	**<0.001**	1.05 (0.94,1.18)	0.377
UA category		0.016		0.985
Low	1.00		1.00	
Medium	**1.23 (1.00,1.52)**	**0.049**	1.02 (0.81,1.28)	0.877
High	**1.52 (1.13,2.04)**	**0.005**	1.03 (0.73,1.44)	0.883

Table shows hazard ratio, 95% CI and *p*-value from Cox regression models

^a^Model 1 is adjusted for sex and age (and age squared). Model 2 is further adjusted for smoking status, BMI, SBP, hypertension meds, cholesterol, HDL cholesterol, ln(triglycerides), lipid meds, diuretic use, ln(CRP) and eGFR

^b^HR is presented for a change of 0.1 mmol/L for UA and for a change of 1.0 for UA Z-score

Multiple UA measurements (range 2–6) were available for a sub-cohort of 2,139 participants whose characteristics did not differ significantly from the full cohort. In this sub-cohort, the average annual change in UA Z-score was 0.0007 per year in those free of CVE and 0.0076 per year in those with known CVE at baseline. For participants free of CVE at baseline, the association between the average UA Z-score and outcomes was very similar to the associations seen for the single UA Z-score in Model 1 and all associations again attenuated in Model 2 (Table 3). The findings in participants with known CVE and multiple UA measurements were also similar to results using one UA measurement (Additional file 1: Table S2).

**Table 3:** Association between UA level and all-cause mortality, CVE mortality and CVE event in the subcohort with multiple UA measures and without history of CVE at baseline

UA measure	Model 1^a^		Model 2^a^	
	HR (95% CI)	*p*-value	HR (95% CI)	*p*-value
All-cause mortality				
UA^b^	**1.17 (1.01,1.36)**	**0.031**	1.17 (0.98,1.40)	0.082
UA z-score^b^	**1.13 (1.00,1.27)**	**0.040**	1.13 (0.98,1.30)	0.097
UA category		0.636		0.906
Low	1.00		1.00	
Medium	1.07 (0.83,1.36)	0.609	1.03 (0.79,1.34)	0.833
High	1.18 (0.84,1.67)	0.343	1.09 (0.73,1.64)	0.658
Average UA z-score^b^	**1.18 (1.02,1.36)**	**0.027**	**1.18 (1.00,1.39)**	**0.046**
Annual change UA z-score^c^	1.14 (0.92,1.41)	0.220	1.11 (0.88,1.39)	0.379
Cardiovascular mortality				
UA^b^	1.18 (0.94,1.49)	0.152	1.10 (0.83,1.47)	0.507
UA z-score^b^	1.14 (0.94,1.37)	0.173	1.07 (0.85,1.35)	0.544
UA category		0.838		0.977
Low	1.00		1.00	
Medium	1.11 (0.75,1.63)	0.603	0.96 (0.63,1.46)	0.841
High	1.15 (0.66,2.00)	0.622	0.98 (0.51,1.89)	0.962
Average UA z-score^b^	1.18 (0.93,1.49)	0.172	1.15 (0.88,1.49)	0.303
Annual change UA z-score^c^	1.21 (0.89,1.64)	0.219	1.10 (0.79,1.54)	0.573
Cardiovascular event				
UA^b^	**1.25 (1.09,1.44)**	**0.001**	1.09 (0.92,1.30)	0.300
UA z-score^b^	**1.17 (1.05,1.31)**	**0.004**	1.05 (0.92,1.20)	0.446
UA category		0.032		0.848
Low	1.00		1.00	
Medium	**1.28 (1.01,1.63)**	**0.042**	1.08 (0.83,1.40)	0.576
High	**1.52 (1.08,2.14)**	**0.015**	1.09 (0.73,1.62)	0.668
Average UA z-score^b^	1.15 (1.00,1.31)	0.053	1.03 (0.88,1.20)	0.746
Annual change UA z-score^c^	**1.31 (1.05,1.64)**	**0.017**	1.11 (0.88,1.41)	0.384

Table shows hazard ratio, 95% CI and *p*-value from Cox regression models

^a^Model 1 is adjusted for sex and age (and age squared). Model 2 is further adjusted for smoking status, BMI, SBP, hypertension meds, cholesterol, HDL cholesterol, ln(triglycerides), lipid meds, diuretic use, ln(CRP) and eGFR

^b^HR is presented for a change of 0.1 mmol/L for UA and for a change of 1.0 for UA Z-score and average UA z-score

^c^HR is presented for a change of 0.1 for annual change in UA Z-score. HR comes from model that also includes average UA z-score

Given the loss of predictive value of UA levels after we adjusted for a large number of confounders in Model 2 we fitted two intermediate models that were the same as Model 2 but which did not adjust for CRP (Model 2A) or did not adjust for SBP/use of antihypertensive drugs (Model 2B). In those free of CVE at baseline, the intermediate model without CRP adjustment (Model 2A in Additional file 1: Table S3) gave similar UA effects to the fully adjusted Model 2, indicating that adjustment for CRP was not responsible for the attenuated UA effect in Model 2. However, the intermediate model that was not adjusted for SBP/antihypertensive drugs (Model 2B in Additional file 1:Table S3) showed similar UA effects to Model 1 indicating that blood pressure was responsible for the attenuated UA effect in Model 2. The results from the intermediate models for the cohort with known CVE at baseline (Additional file 1: Table S4) indicated that in this sub-group neither adjustment for CRP (Model 2A) and/or SBP & anti-hypertensive medications (Model 2B) were responsible for attenuation of the UA effect, compared to the fully adjusted Model 2.

A history of gout was a marginally significant predictor of all-cause mortality in participants with known CVE in a fully adjusted Model 2 (HR 1.65 CI 0.99–2.77) (Table 4). To determine if the effect of UA differed in those with and without a history of gout, we included an interaction between UA and a history of gout in Model 2 and found that the effect of UA on all outcomes was greater in those with a history of gout, but only reached significance for cardiovascular mortality in the group with known CVE (HR 2.13 vs 0.94, *p* = 0.038) (Table 5).

**Table 4:** Hazard ratios (95% CI) for effect of history of gout on outcomes in Model 2 in the full cohort

	CVE history = no		CVE history = yes	
	HR^a^ (95% CI)	*p*-value	HR^a^ (95% CI)	*p*-value
All-cause mortality	0.99 (0.61,1.60)	0.960	1.65 (0.99,2.77)	0.055
Cardiovascular mortality	0.79 (0.36,1.72)	0.553	1.34 (0.63,2.88)	0.447
Cardiovascular event	0.83 (0.48,1.42)	0.492	0.95 (0.56,1.63)	0.863

^a^HR is adjusted for sex, age (and age squared), smoking status, BMI, SBP, hypertension meds, cholesterol, HDL cholesterol, ln(triglycerides), lipid meds, diuretic use, ln(CRP) and eGFR

**Table 5:** Adjusted hazard ratios (and 95% CI) for an increase of 0.1 mmol/L in uric acid overall and by history of gout in the full cohort from an extended Model 2 that includes interaction between history of gout and uric acid level

CVE history = no				CVE history = yes			
Overall (*n* = 3,475)	Gout = yes (*n* = 49)	Gout = no (*n* = 3,426)	*p*-value	Overall (*n* = 698)	Gout = yes (*n* = 25)	Gout = no (*n* = 673)	*p*-value
All-cause mortality							
1.15 (0.98,1.35)	1.65 (0.90,3.03)	1.13 (0.96,1.33)	0.224		1.28 (0.76,2.15)	0.91 (0.75,1.11)	0.214
Cardiovascular mortality							
1.17 (0.91,1.52)	1.77 (0.67,4.69)	1.16 (0.89,1.51)	0.400		**2.13 (1.03,4.43)**	0.94 (0.70,1.24)	**0.038**
Cardiovascular event							
1.09 (0.94,1.26)	1.28 (0.64,2.56)	1.08 (0.93,1.26)	0.638		1.41 (0.91,2.18)	0.95 (0.79,1.13)	0.089

HR is adjusted for sex, age (and age squared), smoking status, BMI, SBP, hypertension meds, cholesterol, HDL cholesterol, ln(triglycerides), lipid meds, diuretic use, ln(CRP) and eGFR

## Discussion

In this prospective study of a well described population in Western Australia the overall prevalence of hyperuricemia was 10.7% which associated with an increased age and gender adjusted risk for incident cardiovascular events and mortality. However adjustments for behavioural and biomedical cardiovascular risk factors completely tempered this effect, except in the subgroup of participants with a history of gout and baseline CVE. The use of time averaged UA levels provided no additional prognostic information.

Our data confirm the complexity of the interactions between UA and the traditional CVE risk factors. The increased CVE risk associated with hyperuricemia (HR 1.52 CI 1.13–2.04, *p* = 0.005) in patients free of CVE at baseline after adjustment for the two most powerful risk factors (age and gender), became non-significant with further adjustment for a range of cardiovascular risk factors (HR 1.03, CI 0.73–1.44, *p* = 0.88). A similar loss of predictive value due to interactions with known risk factors was observed in one of the largest population studies on uric acid (the Framingham Heart study), where mean age at inclusion (47 years) was similar and which also fully adjusted for CVE risk factors [29]. The effect sizes of UA in this study are comparable and in agreement with findings from a large meta-analysis on the relation between UA levels and coronary heart disease where the unadjusted HR of 1.34 decreased to a moderate 1.07 after full adjustment for traditional risk factors [19]. Even in studies where UA was found to predictive of subtypes of CVE, the effect sizes were modest at best [13]. While hyperuricemia cannot be considered a fully independent risk factor for cardiovascular morbidity or mortality in healthy persons, it may still deserve consideration as a risk indicator in the evaluation and possibly management of the metabolic syndrome [13, 30, 31].

Given the notable consistency by which UA can induce CVE risk factors such as hypertension in experimental models [7] we further investigated if certain covariates could be responsible for rendering UA a non-significant predictor. Using intermediate models, that were based on published associations between CVE risk factors and UA, we found that in participants free of CVE at baseline, the effect of UA on future CVE development was lost after adjustment for hypertension. As UA can activate the renin-angiotensin system and is an independent predictor of hypertension incidence and progression in community-based cohorts, this suggests that over time hyperuricemia becomes superseded by hypertension as a main risk factor. This is further supported by findings that a lowering of blood pressure occurs when using uric acid lowering therapies alone [7, 32]. In contrast, in participants with existing CVE, neither the adjustment for CRP or hypertension influenced the UA effect on cardiovascular mortality or other study endpoints. The lack of effect between UA and CRP in patients with established CVE is in opposition with the theory and data that suggest that UA can induce expression of CRP in vascular endothelial and smooth muscle cells [33, 34], where CRP as a marker of low grade vessel inflammation is the important risk factor [35]. It is possible that the results from the intermediate models could be explained by the uric acid paradox theory, i.e. that the role of UA in vascular pathophysiology varies over time and the balance may shift from beneficial (antioxidant) effects of UA towards more pro-atherogenic effects [13, 36–38].

UA levels may fluctuate over time under the influence of both exo- and endogenous factors and higher time averaged UA levels have previously been found to associate with metabolic syndrome [39]. This is one of the first studies to investigate the influence of both the average and change in UA levels over time as a predictor for CVE. While the time averaged UA Z-scores produced more robust hazard ratios in age and gender adjusted models this improved strength was again lost after full adjustment in Model 2. This endorses single measurements instead of averaged UA levels over time in CVE prediction [40].

Hyperuricemia is associated with incident gout in a dose-dependent manner [20, 31, 41]. The prevalence of self-reported doctor diagnosed gout in this population cohort (1.4% in participants free of CVE and to 3.6% in participants with known CVE) fall within the range of published self- and doctor reported gout prevalence rates and confirm its standing as the commonest form of inflammatory joint disease [12, 42–44]. The development of gout is a signal that a critical threshold for uric acid loading has been crossed [45] and the recent introduction of dual energy CT scanning for visualisation and quantification of total body uric acid load could prove to be a powerful tool for delineating this critical threshold [46]. Furthermore, in participants with a history of gout and CVE at baseline an increase of 0.1 mmol/L in UA was independently associated with a more than two-fold increase in risk of cardiovascular mortality. This is in line with several studies showing that gout is a more reliable predictor for CVE and associated mortality than hyperuricemia [11, 21, 22, 31, 41, 45, 47, 48].

Prolonged hyperuricemia or gout increases CVD burden and atherosclerotic plaque development [49] particularly in those with renal impairment [50]. The potential mechanism for the increased risk of serious CVD events, i.e. increased vulnerability of atherosclerotic plaque to rupturing, could be explained by the increase in inflammation about UA crystal deposition in the endothelium, where macrophage activation about the plaque site causes a rupture of the plaque cap in a process similar to cholesterol crystal deposits [2, 51]. Uric acid lowering therapy is therefore likely to have its largest potential to prevent CVE in this particular sub-group.

The prevalence of hyperuricemia in this Western Australian population cohort was considerably lower than the 19% prevalence reported for the same study period in the US based NHANES III study as the prevalence of hyperuricemia has since further risen in the US and was found to be 16% in South Australia between 2008 and 2010 it is more than likely that the WA prevalence has since risen as well [47, 52, 53].

The limitations of this study require consideration. The lower age in participants free of CVE at baseline (as reflected by the low rate of subsequent CVE and death) may have led to an underestimation of the longer term effect of hyperuricemia despite age adjustments. As participants were predominantly of European descent our results cannot be generalised to cohorts with a different ethnic background. Also, the model for the effect of UA on outcomes in people with a history of gout is based on relatively low numbers and should thus be interpreted with caution. Finally, a history of gout was self-reported and we were unable to ascertain the use of uric acid lowering drugs. While we corrected for known risk factors for CVD new methods have been proposed, e.g. Mendelian Randomisation to identify further risk factors (alleles) that contribute to disease process and outcomes in rheumatic diseases [54]. However, we did not have genetic variables available for this cohort. The strengths of this study lie in the long-term prospective follow-up of a large population cohort, the comprehensive baseline data in the complete cohort allowing full adjustment for CVD risk factors. The baseline stratification by the presence of a CV event avoided the cited potential for left-censorship bias in other studies [13]. The validation provided by using multiple uric acid measurements adds to the reliability of our data.

## Conclusions

Despite a significant prevalence in the Western Australian community, there was no attributable cardiovascular risk associated with hyperuricemia in asymptomatic individuals. Our data do suggest that the cardiovascular impact of hyperuricemia varies across the stages of CVE development. Thus, UA lowering therapy for CVE prevention is not warranted in asymptomatic individuals with hyperuricemia, but may benefit patients with gout who have existing CVE.

## Additional file

Additional file 1: Table S1. Association between UA level and all-cause mortality, CVE mortality and CVE event in the full cohort with a history of CVE at baseline. Table shows hazard ratio, 95% CI and p-value from Cox regression models. **Table S2.** Association between UA level and all-cause mortality, CVE mortality and CVE event in the sub-cohort with multiple UA measures and history of CVE at baseline. Table shows hazard ratio, 95% CI and p-value from Cox regression models. **Table S3.** Association between UA level and all-cause mortality, CVE mortality and CVE event in the full cohort without a history of CVE at baseline. Table shows hazard ratio, 95% CI and *p*-value from Cox regression models. **Table S4.** Association between UA level and all-cause mortality, CVE mortality and CVE event in the full cohort with a history of CVE at baseline. Table shows hazard ratio, 95% CI and *p*-value from Cox regression models. (DOCX 38 kb)

## Abbreviations

BHS: Busselton Health Survey
CRP: C-reactive protein
CVD: cardiovascular disease
CVE: Cardiovascular events
EGFR: estimated glomerular filtration rate.
HR: Hazard ratio
ICD: International Classification of Diseases
MSU: monosodium urate
UA: Uric Acid
WA: Western Australia
