# Supplementary material for: Asymptomatic hyperuricemia is not an independent risk factor for cardiovascular events or overall mortality in the general population of the Busselton Health Study
Source: BMC Cardiovasc Disord. 2016 Dec 15;16:256. doi: 10.1186/s12872-016-0421-1 (PMC5160002; doi:10.1186/s12872-016-0421-1)
Supplement: Additional file 1: Table S1. — Association between UA level and all-cause mortality, CVE mortality and CVE event in the full cohort with a history of CVE at baseline. Table shows hazard ratio, 95% CI and p-value from Cox regression models. Table S2. Association between UA level and all-cause mortality, CVE mortality and CVE event in the sub-cohort with multiple UA measures and history of CVE at baseline. Table shows hazard ratio, 95% CI and p-value from Cox regression models. Table S3. Association between UA level and all-cause mortality, CVE mortality and CVE event in the full cohort without a history of CVE at baseline. Table shows hazard ratio, 95% CI and p-value from Cox regression models. Table S4. Association between UA level and all-cause mortality, CVE mortality and CVE event in the full cohort with a history of CVE at baseline. Table shows hazard ratio, 95% CI and p-value from Cox regression models. (DOCX 38 kb) [file 12872_2016_421_MOESM1_ESM.docx]

Additional file 1: Table S1. Association between UA level and all-cause mortality, CVE mortality and CVE event in the full cohort with a history of CVE at baseline. Table shows hazard ratio, 95% CI and p-value from Cox regression models.

|  | Model 1* |  | Model 2* |  |
| --- | --- | --- | --- | --- |
| UA measure | HR (95% CI) | p-value | HR (95% CI) | p-value |
| All-cause mortality | | | | |
| UA** | 1.13 (0.97,1.32) | 0.111 | 0.93 (0.77,1.12) | 0.439 |
| UA z-score** | 1.10 (0.98,1.25) | 0.108 | 0.94 (0.81,1.10) | 0.451 |
| UA category |  | 0.890 |  | 0.153 |
| Low | 1.00 |  | 1.00 |  |
| Medium | 1.07 (0.78,1.47) | 0.667 | 0.83 (0.60,1.16) | 0.277 |
| High | 1.09 (0.74,1.59) | 0.668 | 0.64 (0.41,1.01) | 0.053 |
| Cardiovascular mortality | | | | |
| UA** | **1.26 (1.02,1.57)** | **0.035** | 1.00 (0.76,1.31) | 0.992 |
| UA z-score** | **1.20 (1.00,1.43)** | **0.044** | 0.99 (0.79,1.23) | 0.916 |
| UA category |  | 0.405 |  | 0.626 |
| Low | 1.00 |  | 1.00 |  |
| Medium | 1.09 (0.67,1.75) | 0.734 | 0.79 (0.48,1.30) | 0.351 |
| High | 1.39 (0.81,2.39) | 0.226 | 0.77 (0.41,1.45) | 0.421 |
| Cardiovascular event | | | | |
| UA** | **1.20 (1.05,1.37)** | **0.008** | 0.99 (0.84,1.17) | 0.907 |
| UA z-score** | **1.15 (1.04,1.29)** | **0.009** | 0.99 (0.87,1.13) | 0.874 |
| UA category |  | 0.139 |  | 0.758 |
| Low | 1.00 |  | 1.00 |  |
| Medium | 1.16 (0.87,1.54) | 0.300 | 0.89 (0.66,1.20) | 0.457 |
| High | **1.40 (1.00,1.95)** | **0.048** | 0.91 (0.62,1.35) | 0.653 |

* Model 1 is adjusted for sex and age (and age squared). Model 2 is further adjusted for smoking status, BMI, SBP, hypertension medications, cholesterol, HDL cholesterol, ln(triglycerides), lipid medications, diuretic use, ln(CRP) and eGFR.
** HR is presented for a change of 0.1 mmol/L for UA and for a change of 1.0 for UA Z-score.

Additional file 1: Table S2. Association between UA level and all-cause mortality, CVE mortality and CVE event in the sub-cohort with multiple UA measures and history of CVE at baseline. Table shows hazard ratio, 95% CI and p-value from Cox regression models.

|  | Model 1* |  | Model 2* |  |
| --- | --- | --- | --- | --- |
| UA measure | HR (95% CI) | p-value | HR (95% CI) | p-value |
| All-cause mortality | | | | |
| UA** | 1.13 (0.95,1.33) | 0.162 | 0.92 (0.75,1.14) | 0.450 |
| UA z-score** | 1.09 (0.96,1.25) | 0.185 | 0.93 (0.79,1.10) | 0.404 |
| UA category |  | 0.920 |  | 0.193 |
| Low | 1.00 |  | 1.00 |  |
| Medium | 1.03 (0.73,1.45) | 0.863 | 0.77 (0.54,1.11) | 0.168 |
| High | 1.09 (0.72,1.64) | 0.688 | 0.64 (0.40,1.04) | 0.075 |
| Average UA z-score** | **1.26 (1.04,1.54)** | **0.020** | 1.10 (0.88,1.38) | 0.398 |
| Annual change UA z-score# | 1.16 (0.97,1.38) | 0.103 | 1.01 (0.82,1.23) | 0.928 |
| Cardiovascular mortality | | | | |
| UA** | **1.30 (1.03,1.64)** | **0.025** | 1.09 (0.82,1.46) | 0.544 |
| UA z-score** | **1.23 (1.02,1.48)** | **0.030** | 1.07 (0.84,1.35) | 0.588 |
| UA category |  | 0.442 |  | 0.590 |
| Low | 1.00 |  | 1.00 |  |
| Medium | 1.09 (0.66,1.81) | 0.740 | 0.75 (0.44,1.30) | 0.308 |
| High | 1.40 (0.79,2.49) | 0.247 | 0.82 (0.42,1.58) | 0.550 |
| Average UA z-score** | 1.23 (0.93,1.64) | 0.153 | 1.00 (0.72,1.38) | 0.980 |
| Annual change UA z-score# | **1.32 (1.04,1.68)** | **0.025** | 1.16 (0.87,1.55) | 0.301 |
| Cardiovascular event | | | | |
| UA** | **1.17 (1.01,1.35)** | **0.039** | 0.95 (0.79,1.14) | 0.551 |
| UA z-score** | **1.14 (1.01,1.28)** | **0.033** | 0.96 (0.83,1.11) | 0.608 |
| UA category |  | 0.181 |  | 0.641 |
| Low | 1.00 |  | 1.00 |  |
| Medium | 1.11 (0.81,1.51) | 0.511 | 0.86 (0.62,1.18) | 0.441 |
| High | 1.39 (0.97,2.00) | 0.074 | 0.89 (0.58,1.36) | 0.644 |
| Average UA z-score** | **1.20 (1.01,1.42)** | **0.039** | 1.00 (0.83,1.22) | 0.956 |
| Annual change UA z-score# | 1.11 (0.95,1.30) | 0.192 | 0.93 (0.77,1.12) | 0.438 |

* Model 1 is adjusted for sex and age (and age squared). Model 2 is further adjusted for smoking status, BMI, SBP, hypertension medications, cholesterol, HDL cholesterol, ln(triglycerides), lipid medications, diuretic use, ln(CRP) and eGFR.
** HR is presented for a change of 0.1 mmol/L for UA and for a change of 1.0 for UA Z-score and average UA z-score.
# HR is presented for a change of 0.1 for annual change in UA Z-score. HR comes from model that also includes average UA z-score.

Additional file 1: Table S3. Association between UA level and all-cause mortality, CVE mortality and CVE event in the full cohort without a history of CVE at baseline. Table shows hazard ratio, 95% CI and p-value from Cox regression models.

|  | Model 1* |  | Model 2A* |  | Model 2B* |  | Model 2* |  |
| --- | --- | --- | --- | --- | --- | --- | --- | --- |
| UA measure | HR (95% CI) | p-value | HR (95% CI) | p-value | HR (95% CI) | p-value | HR (95% CI) | p-value |
| All-cause mortality | | | | | | | | |
| UA** | **1.19 (1.04,1.36)** | **0.010** | 1.15 (0.98,1.35) | 0.089 | **1.19 (1.01, 1.39)** | **0.034** | 1.15 (0.98,1.35) | 0.089 |
| UA category |  | 0.210 |  | 0.666 |  | 0.422 |  | 0.666 |
| Low | 1.00 |  | 1.00 |  | 1.00 |  | 1.00 |  |
| Medium | 1.07 (0.86,1.33) | 0.553 | 1.01 (0.79,1.28) | 0.933 | 1.04 (0.81,1.32) | 0.772 | 1.01 (0.79,1.28) | 0.939 |
| High | 1.31 (0.97,1.79) | 0.080 | 1.16 (0.81,1.65) | 0.426 | 1.25 (0.88,1.78) | 0.219 | 1.15 (0.81,1.65) | 0.428 |
| Cardiovascular mortality | | | | | | | | |
| UA** | **1.27 (1.03,1.57)** | **0.025** | 1.17 (0.91,1.52) | 0.225 | 1.27 (0.97,1.65) | 0.079 | 1.17 (0.91,1.52) | 0.225 |
| UA category |  | 0.539 |  | 0.974 |  | 0.747 |  | 0.974 |
| Low | 1.00 |  | 1.00 |  | 1.00 |  | 1.00 |  |
| Medium | 1.16 (0.81,1.67) | 0.411 | 0.99 (0.67,1.47) | 0.964 | 1.06 (0.72,1.58) | 0.756 | 0.99 (0.67,1.47) | 0.964 |
| High | 1.30 (0.79,2.15) | 0.295 | 1.05 (0.58,1.90) | 0.871 | 1.25 (0.70,2.25) | 0.452 | 1.05 (0.58,1.90) | 0.872 |
| Cardiovascular event | | | | | | | | |
| UA** | **1.28 (1.13,1.44)** | **<0.001** | 1.09 (0.94,1.27) | 0.235 | 1.14 (0.98,1.32) | 0.092 | 1.09 (0.94,1.26) | 0.267 |
| UA category |  | 0.016 |  | 0.968 |  | 0.738 |  | 0.985 |
| Low | 1.00 |  | 1.00 |  | 1.00 |  | 1.00 |  |
| Medium | **1.23 (1.00,1.52)** | **0.049** | 1.03 (0.82,1.29) | 0.818 | 1.04 (0.83,1.31) | 0.716 | 1.02 (0.81,1.28) | 0.877 |
| High | **1.52 (1.13,2.04)** | **0.005** | 1.04 (0.74,1.46) | 0.827 | 1.14 (0.81,1.60) | 0.439 | 1.03 (0.73,1.44) | 0.883 |

* Model 1 is adjusted for sex and age (and age squared).
Model 2 is adjusted for sex, age (and age squared), smoking status, BMI, SBP, hypertension medications, cholesterol, HDL cholesterol, ln(triglycerides), lipid medications, diuretic use, ln(CRP) and eGFR.
Model 2A is the same as Model 2 but without adjustment for ln(CRP).
Model 2B is the same as Model 2 but without adjustment for SBP and hypertension medications.
** HR is presented for a change of 0.1 mmol/L for UA.

Additional file 1: Table S4. Association between UA level and all-cause mortality, CVE mortality and CVE event in the full cohort with a history of CVE at baseline. Table shows hazard ratio, 95% CI and p-value from Cox regression models.

|  | Model 1* |  | Model 2A* |  | Model 2B* |  | Model 2* |  |
| --- | --- | --- | --- | --- | --- | --- | --- | --- |
| UA measure | HR (95% CI) | p-value | HR (95% CI) | p-value | HR (95% CI) | p-value | HR (95% CI) | p-value |
| All-cause mortality | | | | | | | | |
| UA** | 1.13 (0.97,1.32) | 0.111 | 0.97 (0.80,1.17) | 0.724 | 0.94 (0.78,1.14) | 0.546 | 0.93 (0.77,1.12) | 0.439 |
| UA category |  | 0.890 |  | 0.302 |  | 0.186 |  | 0.153 |
| Low | 1.00 |  | 1.00 |  | 1.00 |  | 1.00 |  |
| Medium | 1.07 (0.78,1.47) | 0.667 | 0.92 (0.66,1.28) | 0.625 | 0.85 (0.61,1.18) | 0.329 | 0.83 (0.60,1.16) | 0.277 |
| High | 1.09 (0.74,1.59) | 0.668 | 0.72 (0.46,1.12) | 0.145 | 0.66 (0.42,1.03) | 0.068 | 0.64 (0.41,1.01) | 0.053 |
| Cardiovascular mortality | | | | | | | | |
| UA** | **1.26 (1.02,1.57)** | **0.035** | 1.08 (0.83,1.41) | 0.572 | 1.02 (0.78,1.34) | 0.898 | 1.00 (0.76,1.31) | 0.992 |
| UA category |  | 0.405 |  | 0.991 |  | 0.714 |  | 0.626 |
| Low | 1.00 |  | 1.00 |  | 1.00 |  | 1.00 |  |
| Medium | 1.09 (0.67,1.75) | 0.734 | 0.97 (0.59,1.61) | 0.911 | 0.82 (0.50,1.35) | 0.437 | 0.79 (0.48,1.30) | 0.351 |
| High | 1.39 (0.81,2.39) | 0.226 | 0.96 (0.51,1.81) | 0.897 | 0.80 (0.43,1.49) | 0.479 | 0.77 (0.41,1.45) | 0.421 |
| Cardiovascular event | | | | | | | | |
| UA** | **1.20 (1.05,1.37)** | **0.008** | 1.01 (0.86,1.19) | 0.891 | 1.00 (0.85,1.19) | 0.960 | 0.99 (0.84,1.17) | 0.907 |
| UA category |  | 0.139 |  | 0.927 |  | 0.786 |  | 0.758 |
| Low | 1.00 |  | 1.00 |  | 1.00 |  | 1.00 |  |
| Medium | 1.16 (0.87,1.54) | 0.300 | 0.94 (0.70,1.27) | 0.700 | 0.90 (0.67,1.21) | 0.487 | 0.89 (0.66,1.20) | 0.457 |
| High | **1.40 (1.00,1.95)** | **0.048** | 0.96 (0.66,1.42) | 0.853 | 0.92 (0.62,1.35) | 0.666 | 0.91 (0.62,1.35) | 0.653 |

* Model 1 is adjusted for sex and age (and age squared).
Model 2 is adjusted for sex, age (and age squared), smoking status, BMI, SBP, hypertension meds, cholesterol, HDL cholesterol, ln(triglycerides), lipid meds, diuretic use, ln(CRP) and eGFR.
Model 2A is the same as Model 2 but without adjustment for ln(CRP).
Model 2B is the same as Model 2 but without adjustment for SBP and hypertension meds.
** HR is presented for a change of 0.1 mmol/L for UA.
